# An Investigation of Spiritual Well-Being of Individuals with Cancer in Türkiye

**DOI:** 10.1007/s10943-025-02356-w

**Published:** 2025-06-12

**Authors:** Büşra Kurt, Ozlem Sinan

**Affiliations:** 1 Medical Oncology Service, Dr. Abdurrahman Yurtaslan Ankara Oncology Training and Research Hospital, Ankara, Turkey; 2 Department of Midwifery, Faculty of Health Sciences, Marmara University, Istanbul, Turkey

**Keywords:** Spiritual well-being, Nursing, Cancer patient

## Abstract

In illnesses such as cancer, which require prolonged and intensive treatment, patients’ spiritual well-being plays a critical role in enhancing their sense of life meaning and may positively influence treatment outcomes. This descriptive study evaluated the spiritual well-being of patients with cancer. The sample consisted of 300 cancer patients receiving care in the medical oncology service and outpatient chemotherapy unit of a training and research hospital in Ankara between February and May 2024. Data were collected using an introductory information form and the Spiritual Well-Being Scale. A statistically significant relationship was found between the subscale scores and patients’ gender, education level, income level, diagnostic status, and time since diagnosis (*p* < 0.05). In conclusion, the study showed that cancer patients demonstrated moderate levels of meaning and peace and high levels of belief. These findings indicate a potential need for interventions that address the spiritual and religious care needs of cancer patients following diagnosis.

## Introduction

Cancer is a global health concern that profoundly affects multiple dimensions of an individual’s life—social, psychological, physical, and spiritual—and requires long-term treatment and care due to its complex and multifaceted symptomatology (Şentürk et al., 2018). It is the second leading cause of death worldwide, accounting for approximately one in six deaths (WHO, 2022). According to the Global Cancer Observatory data, the global prevalence of cancer is projected to rise by 47%, reaching an estimated 28.8 million cases by 2040 (GLOBOCAN, 2020), highlighting the growing prevalence of cancer as a life-threatening condition associated with acute and chronic side effects, diminished patient functionality, and significant emotional challenges. The disease also contributes to a range of psychosocial issues, including moral distress and depression (Bag, 2013).

Turkiye’s historical and cultural background, deeply rooted in Islamic traditions, shapes how its people perceive spirituality, distinguishing it from Western conceptualizations. Although its administrative system has been structured according to Western standards since the establishment of the Republic, spirituality is primarily understood within the framework of Islamic principles (Dinçer et al., 2021). In Türkiye, religion is often perceived as a structured system of worship and institutionalized practices, whereas spirituality is regarded as an intrinsic force that gives meaning to life, enabling individuals to discover their beliefs and values. Both religion and spirituality play a crucial role in strengthening an individual’s connection to social life by fostering healthy interpersonal relationships. Therefore, understanding the relationship between religion and spirituality is particularly significant in the context of healthcare services (Ekşi & Kaya, 2016).

Spiritual well-being is the connection individuals establish with others, their ability to find meaning and purpose in life, and their belief in and connection to a higher power (Jafari et al., 2010). This dimension of well-being can be adversely affected by illnesses, particularly life-threatening conditions like cancer (Hallaç & Öz, 2011). Factors like changes in body image, altered family roles, and uncertainties about the future significantly impact spiritual well-being during this challenging period (Devi & Fong, 2019). For cancer patients, spiritual well-being serves as a vital source of resilience, enhancing their ability to cope with the disease, fostering hope, improving quality of life, and positively influencing the disease process by alleviating anxiety, stress, depression, and thoughts related to death or suicide (Yılmaz & Yazgı, 2020). Consequently, identifying and addressing the spiritual needs of individuals with cancer is essential to help them navigate the complexities of their condition effectively (Hodge et al., 2014).

Research indicates that spirituality is a critical factor influencing the quality of life in long-term illnesses such as cancer. Consequently, it is recommended that spiritual care be provided to patients as a means of enhancing their levels of spiritual well-being. Furthermore, integrating spiritual well-being as a strategic approach has been suggested to improve the overall quality of life in these patients (AlNatour et al., 2017; Martoni et al., 2017).

Patients with higher levels of spiritual well-being have been observed to experience better quality of life, enhanced coping mechanisms with cancer, and reduced levels of depression and anxiety (Bai & Lazenby, 2015; Puchalski, 2012). Dalcalı et al. (2021) reported that individuals undergoing outpatient chemotherapy exhibited high levels of spiritual well-being. Similarly, Yılmaz and Cengiz (2020) observed high spiritual well-being among cancer patients. In contrast, other studies have found low levels of spiritual well-being in individuals with cancer (Cheng et al., 2019; Merath et al., 2020).

As the spiritual well-being of cancer patients increases, fatigue and pain levels decrease, blood pressure stabilizes, and sleep quality improves (Whitford et al., 2008). Additionally, there is a significant inverse relationship between spirituality and hopelessness or depression, suggesting that higher levels of spirituality are associated with reduced hopelessness and depression among cancer patients (Bernard et al., 2017; Jafari et al., 2010). Spirituality, therefore, forms an integral component of holistic care for cancer patients. Nurses play a crucial role in the provision of spiritual care, supporting patients in developing effective coping mechanisms and planning care tailored to their spiritual well-being (Caldeira et al., 2017; Çınar & Eti Aslan, 2017; Gönenç et al., 2016). Consequently, assessing and addressing the spiritual well-being of cancer patients is essential. The research questions guiding this study are:

- What is the level of spiritual well-being among individuals diagnosed with cancer?
- Is there a relationship between the socio-demographic characteristics of individuals diagnosed with cancer and their spiritual well-being?

## Methodology

This descriptive study evaluated the spiritual well-being of cancer patients. The study population consisted of 1800 patients who attended the medical oncology service and outpatient chemotherapy unit of a training and research hospital in Ankara between February 1 and May 31, 2024. The sample size was determined using G-Power 3.1 for power analysis, calculating a required sample of 265 participants with a 95% confidence interval and a 5% margin of error. Ultimately, the study was conducted with 300 patients who voluntarily agreed to participate.

The study was conducted in a medical oncology clinic and an outpatient chemotherapy unit. The medical oncology clinic has 25 beds, with four patients hospitalized in each room. The outpatient chemotherapy unit, consisting of three sections, features 64 seats, allowing patients to receive treatment between 08:00 and 17:00. In contrast, the medical oncology clinic provides around-the-clock care for patients undergoing long-term chemotherapy or supportive treatments. Additionally, the hospital includes a spiritual support unit staffed by a religious officer assigned by the Ministry of Religious Affairs. However, the staff in this unit does not follow a regular program for patient support.

The inclusion criteria for the study were as follows: being 18 years of age or older, receiving outpatient chemotherapy, receiving treatment in the medical oncology service, and agreeing to take part in the study. Exclusion criteria included individuals with concomitant cognitive or perceptual health issues, as well as those with general condition disorders (oral intake disorders, constipation, diarrhea, nausea and vomiting, tracheostomy, or deep wounds in the mouth).

For data collection, two instruments were utilized: the introductory information form and the Spiritual Well-Being Scale. The introductory information form was developed by reviewing relevant literature (Aktaş & Uğur, 2023; Aktürk et al., 2017; Cankurtaran et al., 2008; Dalcalı et al., 2021) and included 25 questions regarding the socio-demographic characteristics of cancer patients, their engagement with spiritual support, their diagnosis, and their daily life activities.

The Spiritual Well-being Scale was developed by Aktürk et al. (2017) to determine the spiritual well-being of individuals with cancer or other chronic diseases. The scale with three sub-dimensions (peace, meaning, and belief) examines all components of spiritual well-being. It is a Likert-type scale with 12 items (0–None, 1–Very little, 2–Somewhat, 3–Quite, and 4–Very much). The total score on the scale is 0–48 points, and a higher scale score indicates better spiritual well-being. The Cronbach’s alpha value of the scale was 0.87, 0.78 for the meaning subscale, 0.81 for the peace subscale, and 0.93 for the belief subscale. The Cronbach alpha value of the scale was reported as 0.87 in the original study (Aktürk et al., 2017) and 0.81 in this study.

The data collection tools were administered face-to-face at times convenient for the inpatients in the oncology clinic and during the waiting period for patients in the outpatient chemotherapy unit while their treatments were being prepared. For hospitalized patients in the oncology clinic, data collection was generally conducted outside of daytime working hours, when visitors were not present in the room. In the outpatient chemotherapy unit, data were collected by visiting each patient’s chair individually while they awaited their treatments. Data collection tools took approximately 20–30 min.

The institutional permissions and ethics committee approval were obtained from the XXX Ethics Committee (research code 2023-413, dated 06.11.2023). During the implementation phase of the study, the purpose of the study was explained to the cancer patients, and their written informed consent was obtained.

The data were analyzed using the SPSS 21 software package. The socio-demographic characteristics served as independent variables, while the spiritual well-being scale scores were the dependent variables. To determine whether the spiritual well-being scale scores followed a normal distribution, a normality test was conducted using the Shapiro–Wilk test before evaluation. The data were analyzed using descriptive statistics, including numbers, medians, minimum and maximum values, and percentages. As the data did not show a normal distribution, the Mann–Whitney U test was used for comparisons between paired groups, and the Kruskal–Wallis H test was employed to compare three or more groups. The significance level for the analysis results was set at *p* < 0.05.

## Results

Among the patients included in the study, 33% were aged between 51 and 60 years, and 31% were over 61. Most patients were female (56.7%), and a significant proportion (70.7%) were married. In terms of educational background, 34.7% of the patients had completed primary school, and 80% were not employed (Table 2). Furthermore, almost all the patients (92.3%) were receiving chemotherapy as part of their treatment (Table 1).

**Table 1:** Socio-demographic characteristics of patients

Socio-Demographic Characteristics *N* = 300	*N*	%
*Age (56)*		
18–40	43	14.3
41–50	65	21.7
51–60	99	33
61 +	93	31
*Gender*		
Male	130	43.3
Female	170	56.7
*Marital Status*		
Married	212	70.7
Single	88	29.3
*Education level*		
Primary school and below	104	34.7
Secondary school	68	22.7
High school	61	20.3
University	67	22.3
*Employment status*		
No	240	80
Yes	60	20
*Income level*		
Income less than expenses	130	43.3
Income equal to expenses	142	47.3
Income more than expenses	28	9.3
*Social Security*		
Yes	277	92.3
No	23	7.7
*Diagnosis*		
Lung ca	64	21.3
Breast ca	95	31.7
Colon ca	32	10.7
Stomach ca	23	7.7
Connective and soft tissue ca	11	3.7
Liver ca	12	4
Testicular ca	15	5
Over ca	15	5
Rectum ca	14	4.7
Others	19	6.3
*Diagnosis time*		
Less than 1 year	184	61.3
1–3 years	79	26.3
4–6 years	22	7.3
7–9 years	9	3
10 years and over	6	2
*The current treatment for cancer*		
Chemotherapy	277	92.3
Radiotherapy	13	4.3
Surgery	10	3.3

Most patients (80%) had not previously discussed spiritual issues with healthcare personnel. Among those who had, 70% reported discussing with a nurse and 30% with a doctor. Furthermore, 59.3% of patients wanted their caregivers involved in their treatment process. As for their sources of spiritual support, 89.7% preferred family and friends, while 30.3% favored support from nurses, doctors, and other healthcare professionals. Additionally, 54.7% needed to discuss their problems with someone, 52% sought support to alleviate negative thoughts, and 87% needed to feel strong (Table 2).

**Table 2:** Patients’ status of receiving spiritual support

Receiving spiritual support *N* = 300	*N*	%
*Have you talked to healthcare personnel about spiritual issues?*		
Yes	60	20
No	240	80
*If yes, who did you talk to?*		
Nurse	42	70
Physician	18	30
*Do you get enough moral support* *from your family or community after your diagnosis?*		
Yes	281	93.7
No	19	6.3
*Do you want people who provide spiritual support to be included in your treatment?*		
Yes	178	59.3
No	122	40.7
*Do you need to talk about your problems with someone?*		
Yes	164	54.7
No	136	45.3
*Do you need psychological support?*		
Yes	143	47.7
No	157	52.3
*Do you need support to reduce your negative thoughts?*		
Yes	144	48
No	156	52
*Do you want to feel powerful?*		
Yes	261	87
No	39	13
*Who in your family provides you with the most moral support?*		
Spouse	148	49.3
Children	71	23.7
Siblings	41	13.7
Parents	31	10.3
Others	9	3

The median meaning subscale score was 11 (min: 4 and max: 16), peace subscale score was 10 (min: 3 and max: 16), and belief subscale score was 13 (min: 2 and max: 16) (Table 3).

**Table 3:** Distribution of the median scores of the patients on the spiritual well-being scale and its subscales

Subscales	*N*	Median	Min. Score	Max. Score
Meaning	300	11.0	4	16
Peace	300	10.0	3	16
Belief	300	13.0	2	16
The Spiritual Well-Being Scale Total Score	300	33.0	12.0	46.0

*Min* Minimum, *Max* Maximum

The median peace subscale score for female patients was 9, while male patients had a median score of 10. The statistical evaluation revealed a significant difference in the median peace subscale scores across genders (*p* < 0.05). The median belief subscale score for female patients was 13, while male patients had a median score of 12. The statistical evaluation revealed a significant difference in the median belief subscale scores across gender (*p* < 0.05) (Table 4).

**Table 4:** Spiritual well-being subscales score medians according to socio-demographic characteristics of patients *N* = 300 (median, min–max)

	Meaning	*P*		Peace		*P*	Belief		*P*
Gender				Z					
Female	11 (4–16)			9 (3–16)			13 (3–16)		
Male	10 (5–16)	−0.664	0.507	10 (4–16)	−2.15	0.031*	12 (2–16)	−2.11	0.034*
Education level				KW					
Primary school and below ^b**^	10.5 (5–16)			9 (3–16)			14 (2–16)		
Secondary school ^b**^	11 (6–16)			10 (3–16)			12.5 (5–16)		
High school^b**^	11 (4–16)	5.2	0.158	10 (3–16) 11.5	11.5	0.009*	13 (3–16)	15.2	0.002*
University^a**^	10 (6–16)			9 (4–16)			11 (4–16)		
Income level expenses ^b**^				KW					
Income less than expenses ^b**^	11 (5–16)			10 (3–16)			13 (2–16)		
Income equal to expenses ^b**^	10 (4–16)	0.751	0.687	10 (3–14)	0.082	0.961	12 (4–16)	7.7	0.021*
Income more than Expense ^a**^	11 (5–14)			9 (4–14)			12 (3–16)		
Diagnosis				KW					
Lung ca ^b**^	10 (5–16)			9 (5–15)			12 (2–16)		
Breast ca ^a**^	11 (4–16)			9 (4–15)			13 (3–16)		
Colon ca ^a**^	11 (6–15)			10 (7–16)			12.5 (7–16)		
Stomach ca ^b**^	10 (6–16)			9 (3–16)			12 (5–16)		
Connective and	11 (9–13)			10 (9–13)			12 (8–16)		
soft tissue ca ^b**^		9.5	0.392		14.8	0.095		23.2	0.0006*
Liver ca ^b**^	12 (7–16)			10 (7–13)			12.5 (8–16)		
Testicular ca ^b**^	10 (7–12)			9 (5–11)			10 (8–5)		
Over ca ^b**^	10 (7–14)			10 (8–13)			12 (5–16)		
Rectum ca ^b**^	10.5 (7–14)			11 (4–16)			13 (6–16)		
Others ^a**^	11 (5–14)			10 (4–15)			14 (9–16)		
Diagnosis time				KW					
Less than 1 year ^a**^	10 (4–16)			9 (4–16)			12 (2–16)		
1–3 years ^a**^	11 (6–16)			10 (3–16)			12 (4–16)		
4–6 years ^b**^	10 (7–15)	5.8	0.215	10 (4–16)	3.03	0.553	15 (3–16)	12.6	0.013*
7–9 years ^b**^	12 (8–16)			12 (8–15)			15 (12–16)		
10 years and over ^b**^	10 (8–12)	9.5 (6–12)		15.5 (5–16)			15 (3–16)		

*p < 0.05, **a > b, KW = Kruskal–Wallis Test, Z = Mann–Whitney U Test

The median peace subscale score for patients with a university degree and primary school degree or less was 9, while it increased to 10 for those with a secondary school degree, and high school degree. The statistical evaluation revealed a significant difference in the median scores of the peace subscale according to the patients’ educational status (*p* < 0.05). The median belief subscale score for patients with a university degree was 11, while it increased to 12.5 for those with a secondary school degree, 13 for those with a high school degree, and 14 for those with a primary school degree or less. The statistical evaluation revealed a significant difference in the median scores of the belief subscale according to the educational status (*p* < 0.05) (Table 4).

The median belief subscale score for patients with an income more than expenses and equal to expenses was 12, while it increased to 13 for those with an income less than expenses. A significant difference was found in the median scores of the belief subscale according to the patients’ income level (*p* < 0.05) (Table 4).

The table shows that the belief subscale score medians of patients with a diagnosis of colon cancer (12.5), breast cancer (12), and other cancers (14) are higher than the score medians of patients with lung cancer (12), stomach cancer (12), testicular cancer (10), and ovarian cancer (12). There was a significant difference in the median scores of belief subscale based on the patients’ diagnoses (*p* < 0.05) (Table 4).

The belief subscale score medians of patients with diagnosis time less than 1 year (12) and 1–3 years (12) are lower than those with 4–6 years (15), 7–9 years (15) and 10 years and over (15.5). There was a significant difference in the median scores of the belief subscale based on the diagnosis time (*p* < 0.05) (Table 4).

## Discussion

This study examined the spiritual well-being of individuals with cancer using the “Spiritual Well-Being Scale (SWBS).” The Spiritual Well-Being Scale (SWBS), while widely used and validated for assessing spiritual well-being, has been criticized in the literature for bias due to potential contamination. As a result, analyses were conducted separately on its subscales to address this concern (Koenig & Carey, 2024, 2025).

The median belief subscale score was higher than the meaning and peace subscale score. The median meaning and peace subscale score was determined to be at a moderate level. Muzdalifah et al. (2022), Aktürk et al. (2017) and Martoni et al. (2017) reported that patients received the highest score from the meaning subscale in their studies conducted with cancer patients, while Pearce et al., (2012) reported that patients received low scores from the meaning and peace subscale in their study examining the spiritual well-being of cancer patients. Kavalalı Erdoğan and Koç (2021) reported that adult oncology patients received the highest score from the meaning, peace and faith subscale in their study. Guz et al. (2012) reported that Turkish oncology patients often benefit from religious treatments. In the same study, participants often resorted to prayer to fight illness and carried certain healing verses to receive hope and peace. When individuals have an optimistic and strong spiritual view of life, they can cope more effectively; prayer, hope and trust make it easier to cope with illness (Kavalalı Erdoğan & Koç, 2021).

The median scores of the patients’ belief subscale was high in the study. Individuals diagnosed with cancer tend to exhibit high levels of belief subscale (Dalcalı et al., 2021; Muzdalifah et al., 2022). These findings are consistent with our study, which also revealed a high level of spiritual well-being among cancer patients. In the meta-analysis study conducted by Salsman et al. (2015), it was explained that religion and beliefs play an important role in regulating the emotional state in life threats like cancer. The high level of belief among patients also suggests that individuals diagnosed with cancer are more likely to turn to spirituality to cope with the disease.

In the study, one of the characteristics that differed in the subscales of the patients was gender. In the peace subscale, the median scores of men were higher than those of women. In the belief subscale, the median scores of women were higher than those of men. Martoni et al. (2017) reported a statistically significant difference indicating that women had higher average faith subscale scores than men. Piderman et al. (2015) found that spiritual well-being scores were lower in men than women.

Education level significantly influenced patients’ peace and belief subscale, with higher education levels associated with lower peace and belief levels. This finding aligns with the results of Dalcalı et al. (2021) and Pahlevan and Ong (2019), which demonstrated that lower education levels were associated with greater spiritual orientation. Conversely, Caldeira et al. (2017) reported that patients with higher education levels experienced less spiritual distress, highlighting contrasting perspectives in the literature. The study revealed a notable finding that scores on the peace and belief subscales decreased with increasing education levels among individuals diagnosed with cancer. This suggests that spiritual and optimistic feelings may hold greater significance for patients with lower educational attainment, potentially reflecting a greater reliance on spirituality as a coping mechanism during the disease process.

The study identified income level as a significant factor influencing patients’ belief subscale scores, with higher income levels associated with lower levels of belief. Cancer patients often face substantial treatment-related expenses; thus, better economic conditions may offer greater support and reduce financial stress. This reduction in stress may, in turn, lessen the reliance on faith-based coping mechanisms (Muzdalifah et al., 2022).

Another factor influencing patients’ belief subscale score was their primary diagnosis. Patients with colon, breast, or other types of cancer had higher levels of belief subscale, while those with other types of cancer showed moderate to good levels of belief subscale. In a study by Cheng et al. (2023), the spiritual well-being scores of patients with stomach and colon cancer were moderate. Similarly, studies by Kahraman and Pehlivan (2023) on lung cancer patients and Jafari et al. (2013) on breast cancer patients reported moderate or good levels of spiritual well-being. These findings are consistent with the results of the present study, indicating that belief becomes more important for patients following their diagnosis. Cancer is a life-threatening illness and is commonly associated with death. Cancer patients may need to establish a relationship with God to cope with their fear of death and their need for support (Taylor, 2006). Furthermore, the belief subscale scores of patients improved as the duration of the diagnosis time increased. Kahraman and Pehlivan (2023) reported in their study on patients with lung cancer that early-stage disease was a factor negatively affecting spiritual well-being scores. These results suggest that patients may initially experience emotions such as shock, denial, anger, fear, and sadness upon diagnosis, as time passes, to manage the disease process patients’ belief improves. Individuals may tend to be more religious when coping with the disease (Rippentrop, 2005).

## Limitations

The study has some limitations. Data were collected from cancer patients receiving care in the medical oncology service and outpatient chemotherapy unit of a training and research hospital in Türkiye. As the findings were derived from a single institution, they may not be generalizable to the broader population of cancer patients.

## Conclusion

In conclusion, the study demonstrated that individuals with cancer generally exhibited moderate levels on the meaning and peace subscales and high levels on the belief subscale, highlighting the significant role of religion in coping with the challenges associated with cancer. Nurses play a crucial role in addressing the spiritual care and religious needs of cancer patients throughout their diagnosis, treatment, and recovery processes. They can inform patients about people who may help with spiritual or religious needs. It is recommended that nursing education curricula incorporate courses on spirituality, and spiritual care, utilizing effective teaching methods to enhance understanding and application and nurses be encouraged to recognize and reinforce patients’ spiritual strengths and support them in utilizing these resources as part of comprehensive care.
